# Contrasting patterns of leaf trait variation among and within species during tropical dry forest succession in Costa Rica

**DOI:** 10.1038/s41598-017-18525-1

**Published:** 2018-01-10

**Authors:** Géraldine Derroire, Jennifer S. Powers, Catherine M. Hulshof, Luis E. Cárdenas Varela, John R. Healey

**Affiliations:** 1 Bangor University, School of Environment, Natural Resources and Geography, Bangor, LL57 2UW UK; 2 Swedish University of Agricultural Sciences, Southern Swedish Forest Research Centre, Alnarp, 23053 Sweden; 3 University of Minnesota, Department of Ecology, Evolution & Behavior and Department of Plant and Microbial Biology, Saint Paul, MN 55108 USA; 4 Universidad de Puerto Rico Mayagüez, Departamento de Biología, Mayagüez, PR 00681-9000 Puerto Rico; 5 Universidad Nacional de Costa Rica, Laboratorio de Ecología Funcional y Ecosistemas Tropicales, Escuela de Ciencias Biológicas, Heredia, 86-3000 Costa Rica

## Abstract

A coordinated response to environmental drivers amongst individual functional traits is central to the plant strategy concept. However, whether the trait co-ordination observed at the global scale occurs at other ecological scales (especially within species) remains an open question. Here, for sapling communities of two tropical dry forest types in Costa Rica, we show large differences amongst traits in the relative contribution of species turnover and intraspecific variation to their directional changes in response to environmental changes along a successional gradient. We studied the response of functional traits associated with the leaf economics spectrum and drought tolerance using intensive sampling to analyse inter- and intra-specific responses to environmental changes and ontogeny. Although the overall functional composition of the sapling communities changed during succession more through species turnover than through intraspecific trait variation, their relative contributions differed greatly amongst traits. For instance, community mean specific leaf area changed mostly due to intraspecific variation. Traits of the leaf economics spectrum showed decoupled responses to environmental drivers and ontogeny. These findings emphasise how divergent ecological mechanisms combine to cause great differences in changes of individual functional traits over environmental gradients and ecological scales.

## Introduction

The functional trait approach has gained momentum in the last two decades for the study of individual plant fitness and the assembly of communities along environmental gradients^1^. Most functional trait studies are based on the two assumptions that most of the variation in trait values occurs between species^2^, and that traits are coordinated within plant strategies^3^. These two assumptions are increasingly challenged and their validity across spatial, temporal and ecological scales is debated^4–6^.

Most functional trait-based studies overlook the variation of trait values occurring within species by using mean values per species calculated from a limited number of individuals^7^, even though functional traits are defined as features measured at the scale of individuals. However, this approach is now challenged^7^, as there is increasing evidence that intraspecific trait variation (ITV) is not negligible compared with interspecific variation^8^, including in tropical forests^9,10^, and patterns observed within and between species can differ^11^. Moreover, ITV can represent an important form of community response to environmental gradients^5,12–14^. ITV results from both genetic variation among individuals and the ability of a genotype to express different phenotypes (phenotypic plasticity). Both these mechanisms can be a response to environmental conditions and they are difficult to disentangle without experimental studies^7^. The phenotype expressed by a given genotype can also change with the development of a plant (*i*.*e*. ontogeny)^15^. The importance of including ITV in functional trait studies is still openly debated and depends on the spatial^4,5^ and ecological^6^ scales, and traits, considered^16^.

General patterns of correlations and trade-offs among plant functional traits allow suites of correlated traits to be interpreted as plant ecological strategies^1^. The leaf economics spectrum, one of the most studied axes of trait associations, describes the range from (1) acquisitive strategies with fast return on investment of resources in leaves characterized by high specific area, nutrient concentrations, metabolic rates and short lifespan, to (2) conservative strategies with a slow return on investment characterized by low metabolic rates and expensive leaf construction and defence costs allowing a longer lifespan^1,3^. The validity of this pattern among species at the global scale has been demonstrated using trait databases^3^. However, its validity at finer scales is an emerging question: correlated traits can differ in their sensitivity to genetic variation, environmental gradients and ontogeny^6^, and observed patterns in interspecific variation do not necessarily hold within species^17^. There is therefore a need to improve knowledge of how trait covariations hold across spatial, temporal and ecological scales^6^.

Our study aims to understand the importance of ecological scales and environmental changes in the patterns of trait variation and covariation in the context of tropical dry forest succession. Successional forests undergo large environmental changes with canopy closure^18^. As has been observed along other gradients^12,13,19^, these environmental changes are likely to affect functional traits at different ecological scales (intra- and inter-specific) and changes in community trait values can result from both species turnover and ITV. However, most studies of functional trait changes during forest succession have focused on interspecific changes and we are unaware of any previous study considering ITV during succession. In tropical wet forests, the decrease in light availability leads to a change from acquisitive to conservative strategies during succession^20,21^. In tropical dry forests (TDF), the light gradient during succession is less pronounced^22^, and the decreases in water deficit and temperature may be stronger drivers of change in plant communities^18,23^. Because conservative strategies are advantageous in dry conditions^1^, the increase in water availability during succession in TDF predicts a change from conservative to acquisitive strategies. This hypothesis has received mixed support from field studies: for example, trends in specific leaf area vary among studies^20,23–26^. Moreover, some studies found that leaf phenological habit and morphological traits allowing leaf cooling and water status control are more important than leaf economics strategies^21,23^ in successional TDF. The directionality of changes in leaf traits among species in successional TDF remains unresolved, and the changes through ITV are still largely unexplored (although Falcão *et al*.^27^ found that they are directional in one species in Brazilian TDF). At the scale of individual trees, changes in functional trait values can result from environmental changes during succession, the smaller-scale heterogeneity in light levels within the forest, and ontogenetic changes. Assessing if these changes lead to similar or opposite trends, and hence reinforce or oppose each other, is important to understand the trajectories of communities during succession. A decrease with ontogeny and increasing light availability in trait values associated with acquisitive strategies has been observed in temperate^28–30^ and tropical wet forests^31,32^ but, to the best of our knowledge, this has not yet been studied in TDF.

To contribute to understanding of the effect of scale and environmental gradients on functional trait changes in successional TDF, our study considers different ecological scales (community scale and individual scale, and variation between and within species) during the relatively long temporal scale of forest succession. We studied sapling communities in two forest types differing in physical environment, to distinguish idiosyncratic from more general patterns. We expanded on previous studies of changes in leaf traits through secondary succession in tropical forests by measuring leaf traits on a large sample of individuals allowing thorough study of two major contributors to ITV: the effect of environmental changes due to succession and local light heterogeneity, and the effect of ontogeny. We focused on early ontogenetic stages (saplings) because of their importance for tree establishment and community assembly and dynamics^31^, while still considering a large ontogenetic window within the sapling stage. Our study is the first to simultaneously evaluate changes through species turnover and ITV in response to the overall environmental gradients during succession and to ontogeny in tropical forest. We asked: (1) what are the relative contributions of species turnover and ITV to changes in community leaf trait values during succession and are they consistent among traits? We expected the contribution of ITV to be lower than that of species turnover but still a notable component. We also expected these relative contributions to differ among traits because trait correlations observed at interspecific scales may not hold within species. (2) How do plant strategies in sapling communities change during succession? We expected a trend from conservative to acquisitive strategies. However, this trend may not be consistent for all traits of the leaf economics spectrum because these traits can have different sensitivities to environmental gradients. We also expected differences between the two forest types in the trends of change in functional composition with the successional gradients because of differences in environmental conditions between forest types. (3) What is the direction of changes in leaf traits with sapling ontogeny and light environment when measured at the scale of individual saplings? We proposed two opposing hypotheses regarding ontogenetic changes: ontogeny could select for a trend from acquisitive to conservative strategies as it does in other biomes, or changes through succession and ontogeny could follow the same trend thus maximizing the fitness of an individual throughout its life.

## Results

We surveyed 967 saplings and measured a total of 2539 leaves on 852 saplings of 69 species. The most common families were Fabaceae and Rubiaceae (respectively 21.0% and 13.4% of individuals). Species mean trait values are listed in Supplementary Table S1. As expected^18^, the below-canopy environment became cooler and darker with succession: canopy openness decreased significantly (*P* < 0.05), in both seasons for the forest type dominated by oaks (OAK) and in the dry season for the forest type with a more even mixture of species (MIX). Air temperature, during the dry season for MIX and the wet season for OAK, tended to decrease with succession, although not significantly (0.05 < *P* < 0.1) (Supplementary Table S2). Soil factors were generally not significantly correlated with successional age, which limits the risk of confounding bias. Environmental variables differed between the two forests types: OAK plots had more nutrient-poor soil and harsher microclimatic conditions (higher temperature and canopy openness) (Supplementary Table S2). A Hill-Smith ordination performed on all measured leaves confirmed that the leaf economics spectrum is the main axis of correlation among traits, and differentiated legumes on the second axis (Supplementary Fig. S1). Community mean leaf trait values per forest type are given in Supplementary Table S3.

### Changes with succession – multi-trait analyses

**Figure 1:**
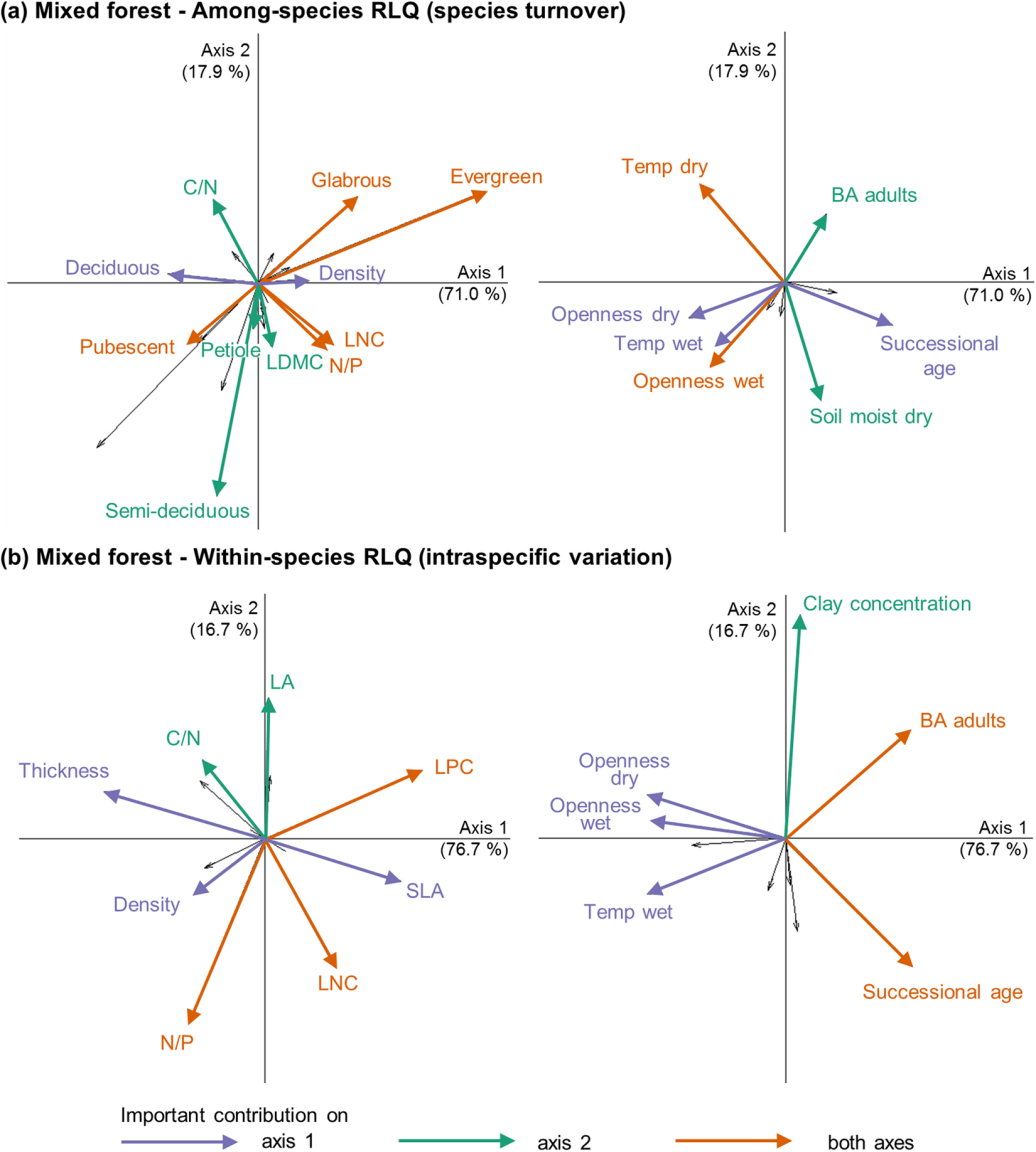
First factorial planes for (**a**) the among-species RLQ and (**b**) the within-species RLQ of the relationship between sapling leaf traits (left plot) and environmental factors (right plot), in mixed forest, performed on 406 individuals of 51 species. For each partial RLQ, traits and environmental factors are presented in the same factorial plane but are plotted separately for ease of reading. The arrows represent the coefficients of each variable in the first factorial plane. Arrows pointing in the same direction represent variables co-varying positively. The labels and arrows in colour indicate traits and environmental factors that contribute more to an axis than if all traits and environmental factors contributed equally (>4.7% for traits and >10% for environmental factors). Contributions and coefficients of traits and environmental factors are presented in Supplementary Table S4. “Petiole” stands for “petiole length”, “Openness” for “canopy openness”, “Temp.” for “air temperature” and “Soil moist.” for soil moisture, and “dry and “wet” indicate the season of measurements of these environmental factors.

**Figure 2:**
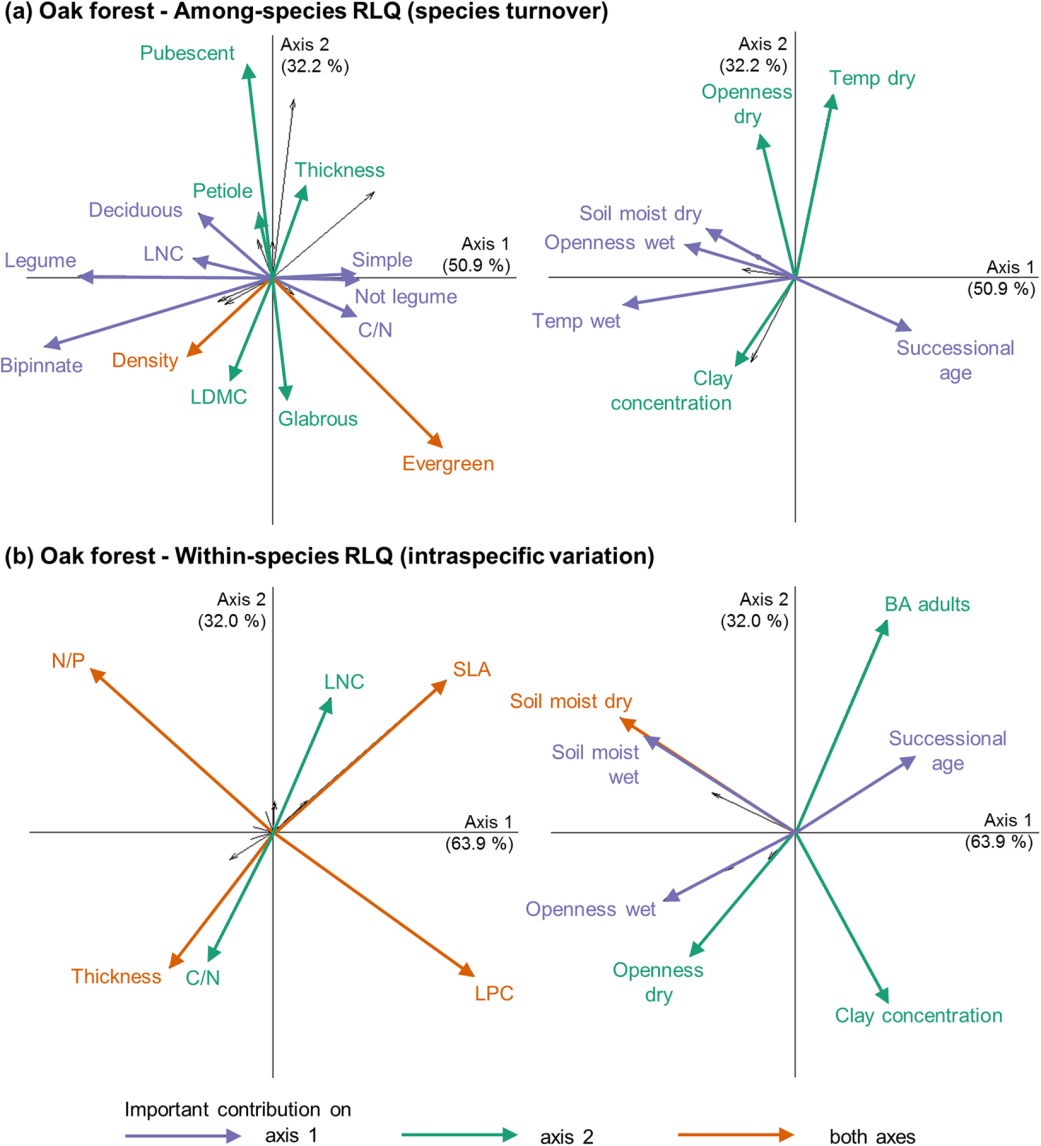
First factorial planes for (**a**) the among-species RLQ and (**b**) the within-species RLQ of the relationship between sapling leaf traits (left plot) and environmental factors (right plot), in oak forest, performed on 553 individuals of 44 species. Details on interpretation and abbreviations are given in Fig. 1.

The RLQ analyses (Figs 1 and 2) showed strong links between sapling traits and environmental factors, with a high proportion of the total cross-covariance explained by the first two axes. Most of the structure was explained by species turnover as shown by the higher eigenvalues of the among-species RLQs (3.62 for the first two axes in MIX and 3.58 in OAK) compared with those of the within-species RLQs (0.27 and 0.53). For both forest types, and for among- and within-species RLQs, the first axis explained most of the variation (71.0% among-species and 76.7% within-species in MIX, and 50.9% and 63.9% in OAK). It opposed successional age at one end of the axis to high canopy openness and air temperature (and high soil moisture for OAK) at the other end. For among-species RLQs, the traits associated with this first axis differed between forest types: in MIX, trait values associated with tolerance of dry and hot conditions (deciduous and pubescent) at the early succession end of the axis were opposed to trait values associated with lower tolerance of these conditions (evergreen and glabrous) and to high leaf density, leaf nitrogen concentration (LNC) and N/P ratio in later succession. In OAK, early succession was associated with trait values characteristics of legumes (legumes, bipinnate and high LNC)^33^, and deciduous and high density leaves, and opposed to simple, evergreen and high C/N ratio leaves, and non-legumes, at the other end of the axis. For within-species RLQs, there was a similar trend for both forest types: trait values associated with acquisitive strategies in late succession (high specific leaf area (SLA) and leaf phosphorus concentration (LPC) for both forest types and high LNC for MIX) opposed to high leaf thickness and N/P for both forest types and leaf density for MIX.

### Changes with succession – single-trait analyses

**Figure 3:**
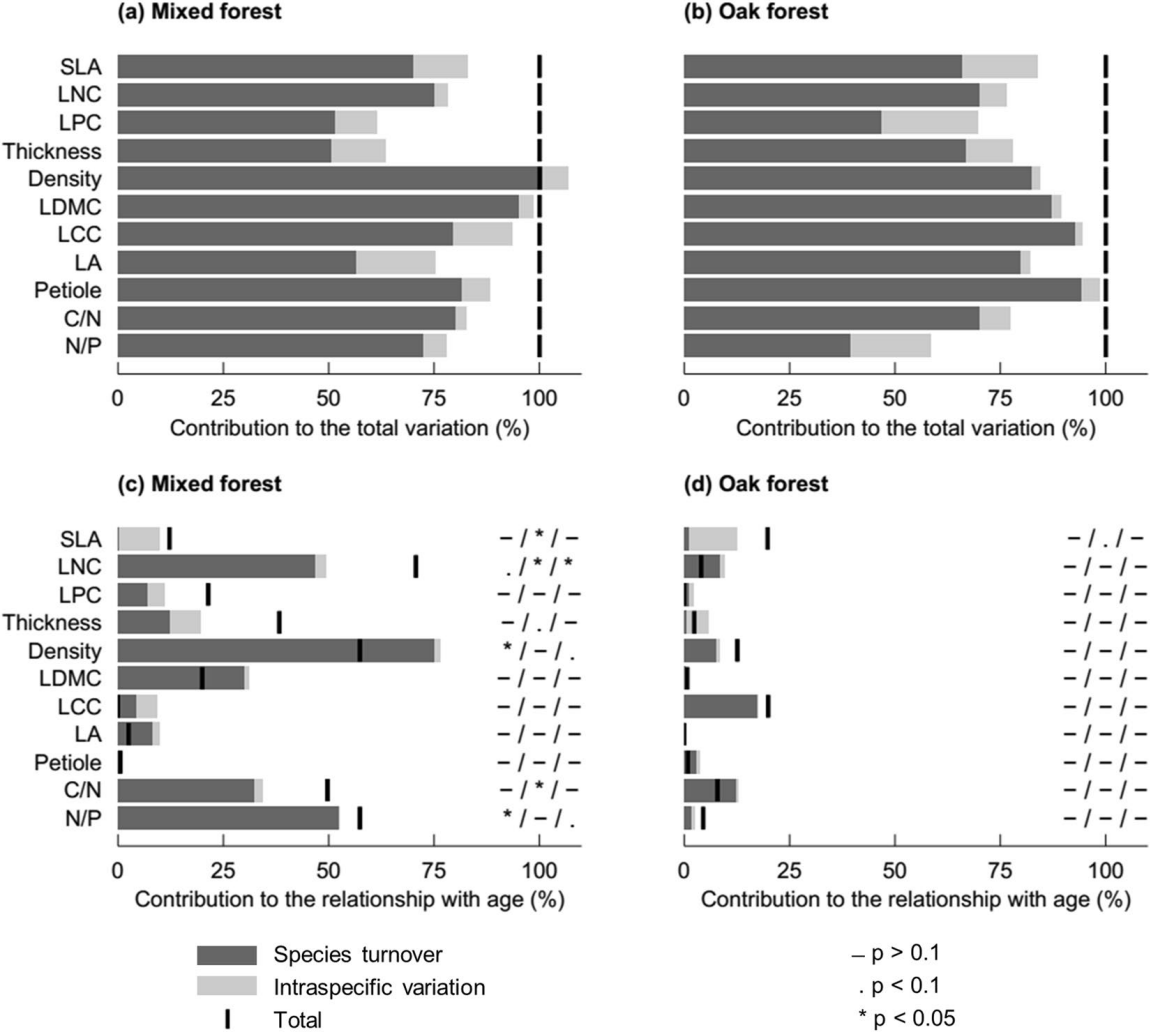
Contributions of species turnover, intraspecific variation and their covariation to the total variation of community trait values (**a**,**b**), and to the relationship between the community trait values and successional age (**c**,**d**), for mixed and oak forest types respectively (analysis performed on six plots for each forest type). Thick black lines show the total contribution of species turnover, intraspecific variation and their covariation. If the covariation is positive the line is on the right of the bar, and if negative on the left. The p-value of the relationship with successional age is given at the right of each bar in this order: species turnover/intraspecific variation/total.

Species turnover contributed more than ITV to the total variation of community trait values (Fig. 3a,b). The traits showing the highest contribution of ITV to the total variation were SLA and leaf thickness for both forest types, leaf area (LA) and leaf carbon concentration (LCC) in MIX and LPC and N/P in OAK.

Across successional age, species turnover contributed more to community trait variation than did ITV, except for SLA (Fig. 3c,d). For all the relationships that were not significant when considering only species turnover, adding ITV (*i*.*e*. considering species turnover and ITV) did not make the relationships significant. The effect of successional age on community trait values differed between the two forest types. SLA was the only trait showing a notable change for both forest types, albeit only significantly for MIX (*P* < 0.05 for MIX, *P* < 0.1 for OAK), and it showed a much higher contribution of ITV than of species turnover. For OAK, no other change was notable. For MIX, some traits changed through ITV but not species turnover (C/N significantly (*P* < 0.05) and leaf thickness not significantly (0.5 < *P* < 0.1)), some traits changed through species turnover but not ITV (leaf density and N/P, both *P* < 0.05), while LNC showed evidence of change through both (*P* < 0.05 for ITV and *P* < 0.1 for species turnover). The directions of these changes (Table 1) suggest a trend from conservative to acquisitive strategies through ITV (decrease in leaf thickness and C/N, and increase in SLA and LNC), and through species turnover (increase in LNC and N/P).

**Table 1:** Slopes (Coef.) and significances (*P*) of the linear regressions between successional age and the changes in community mean trait value due to species turnover only, intraspecific variation (ITV) only, and species turnover and ITV (performed on six plots for each forest type).

**Forest type**	**Trait**	**Species turnover**		**ITV**		**Species turnover and ITV**	
		Coef.	*P*	Coef.	*P*	Coef.	*P*
Mixed forest	Leaf thickness	−0.0005	0.319	**−0.0004**	**0.088**	−0.0008	0.190
	SLA	0.0770	0.926	**0**.**5772**	**0**.**029**	0.6542	0.498
	Leaf density	**0**.**0013**	**0**.**027**	−0.0002	0.373	**0**.**0011**	**0**.**081**
	LNC	**0**.**1905**	**0**.**061**	**0**.**0432**	**0**.**014**	**0**.**2337**	**0**.**036**
	C/N	−0.1324	0.174	**−0.0313**	**0.026**	−0.1637	0.118
	N/P	**0**.**2095**	**0**.**032**	0.0097	0.785	**0.2192**	**0.081**
Oak forest	SLA	0.3831	0.798	**1.1635**	**0.059**	1.5466	0.377

Only traits that have at least one relationship with *P* < 0.1 are presented. Relationships with *P* < *0.1* are highlighted in bold (Supplementary Table S5 gives the value for all traits).

For the traits without recorded ITV (leaf phenological habit, compoundness, pubescence and legumes), the only significant relationship that we found was a decrease in the proportion of saplings of deciduous species with successional age (slope of GLM −0.085, *P* = 0.009, *R* ^2^ = 0.59), which was not dependent on forest type.

### Intraspecific changes with ontogeny and environment

Among the 11 traits measured within species, most (with the exception of LA) showed significant directional changes with at least one of the variables measured at the scale of individual sapling (ratio between the height of the individual and the maximum height of the species (H/Hmax) as a proxy for ontogeny, forest type and crown illumination index (CII)) (Table 2). These changes, although considerably smaller than those among species (as shown by the small *R* ^2^_*M*_ compared with *R* ^2^_*C*_), were not negligible for most of the traits (with the exception of petiole length and LA), and explained up to 18% of the total variance of the model (for SLA, as shown by the *R* ^2^_*M*_). Only three traits changed significantly with ontogeny: leaf thickness and LCC increased, and SLA decreased, which shows a trend from acquisitive to conservative strategies. LCC was the only trait for which the change with ontogeny depended on the forest type (it increased faster in OAK than MIX). Many more traits changed significantly with CII (leaf thickness, leaf dry mater content (LDMC), leaf density, LCC and C/N increased, and SLA, LPC and LNC decreased) and with forest type (higher leaf thickness, LDMC, leaf density, LPC, C/N and lower SLA, LNC, N/P in OAK than MIX). These changes showed an overall trend from more acquisitive and less drought- and heat-tolerant strategies in low-light conditions and in MIX, to more conservative and drought-tolerant strategies in high-light conditions and in OAK.

**Table 2:** Selected models for the intraspecific trait variation at the scale of individual saplings (linear mixed models on log-transformed trait values performed on 415 individuals of 26 species).

Trait	Random part	R^2^_C_	R^2^_M_	H/HMax		Forest type		Crown illumination index		Interaction H/Hmax * forest type	
				Coef.	*P*	Coef.	*P*	Coef.	*P*	Coef.	*P*
Leaf thickness	I	0.71	0.12	**0**.**52**	**<0**.**001**	**−0.08**	**<0.001**	**0**.**08**	**<0**.**001**	—	—
Petiole length	I+S	0.96	<0.01	0.02	0.972	−0.05	0.128	**0**.**07**	**0**.**008**	—	—
LA	I+S	0.88	<0.01	−0.05	0.942	0.02	0.868	0.07	0.062	1.01	0.090
SLA	I+S	0.80	0.18	**−0**.**93**	**0**.**031**	**0.17**	**<0.001**	**−0**.**15**	**<0**.**001**	—	—
LDMC	I+S	0.86	0.08	0.38	0.177	**−0.08**	**<0.001**	**0**.**06**	**<0**.**001**	—	—
Leaf density	I+S	0.80	0.09	0.43	0.161	**−0.09**	**<0.001**	**0**.**07**	**<0**.**001**	—	—
LPC	I+S	0.50	0.04	0.68	0.152	**−0.16**	**<0.001**	**−0**.**06**	**0**.**035**	—	—
LCC	I+S	0.82	0.05	**0**.**14**	**0**.**002**	0.01	0.124	**0**.**01**	**<0**.**001**	**−0**.**07**	**0**.**027**
LNC	I	0.83	0.04	—	—	**0.09**	**<0.001**	**−0**.**03**	**0**.**029**	—	—
C/N	I	0.83	0.04	—	—	**−0.10**	**<0.001**	**0**.**03**	**0**.**004**	—	—
N/P	I	0.46	0.09	—	—	**0.24**	**<0.001**	—	—	—	—

The structure of the random part of the model is indicated by “I” for random intercept only and “I + S” for random intercept and slope of H/Hmax. *R* ^2^_*C*_ is the conditional *R* ^*2*^, *i*.*e*. the variance explained by the whole model. *R* ^*2*^_*M*_ is the marginal *R* ^*2*^, *i*.*e*. the variance explained by the fixed factors only^54^. Significant relationships (*P* < 0.05) are highlighted in bold.

## Discussion

### Relative contributions of species turnover and intraspecific variation to successional changes

We found that the contribution of species turnover to changes in leaf functional traits of sapling communities with succession was more important than the contribution of ITV. Moreover, including ITV did not change the community-level trends observed during succession, contrary to studies across other environmental gradients^12,34^. This low relative contribution of ITV to the change experienced by TDF sapling communities in our study can result from a combination of explanations. (1) The interspecific variation of trait values is generally greater than their intraspecific variation, as shown by our results (Supplementary Fig. S2), by several other studies in TDF^9,33^, and globally^8^. (2) Higher importance of species turnover has been found in steep environmental gradients^14,35^, whereas over short time scales^36^ and small spatial scales^4,34^ the response is more likely to be dominated by plastic adjustments to the phenotype of existing plants than by species replacements, leading to a higher contribution of ITV^36^. Although our study was conducted at a relatively small spatial scale, our chronosequences covered long, decadal timescales (25 and 48 years for the OAK and MIX forest types, respectively), which, in the context of successional forests, leads to a steep gradient of environmental conditions. These long time scales and steep gradients can explain the high species turnover among plots, as shown by the relatively low Sørensen similarity indices between plots of different successional ages (mean 0.409 and range 0.222–0.615 in OAK, and mean 0.335 and range 0.077–0.615 in MIX), leading to the higher contribution of species turnover to the total variation of trait values among plots (Fig. 3a,b). (3) The high species turnover can also result from the large number of species in the regional pool (we found 69 species in our plots alone and Powers *et al*.^37^ surveyed 120 species in the same area), which, combined with the relatively small size of our plots, may lead to the large differences in their specific composition.

Despite its low contribution to changes in community trait values, ITV was associated with the overall environmental gradient during succession (shown by the high percentage of variance explained by the first axes of the within-species RLQs and significant changes observed for four traits in the single-trait analyses). This shows that the filtering effect of environmental conditions on trait values within species found along different environmental gradients^12,13,19^ is relevant in the context of successional gradient in TDF.

In our study, functional traits differed greatly in the relative contribution of species turnover and ITV to their changes during succession (Fig. 3c,d, and RLQs). This result, which is in line with studies of community assembly along other gradients^12,14,34^, confirms that, despite the correlations observed among them (Supplementary Fig. S1), functional traits can differ in their variance structure across ecological scales (intra- *vs*. inter-specific)^6^, leading to a decoupling of trait coordination. SLA showed a high contribution of ITV to the changes along the successional gradient; it is the only trait for which the contribution of ITV exceeded that of species turnover. This result, also observed for other environmental gradients^14,35^, is consistent with the great variation of SLA at the intraspecific scale^6,8,38^. SLA, a composite trait, can change in response to the multiple environmental changes in water and light availability during succession that may affect leaf mass and leaf area^6^. Our study also shows that traits like SLA and LPC, which are strongly coordinated within the leaf economics spectrum (Supplementary Fig. S1) and show a high contribution of ITV to their overall variation (Fig. 3a,b) when considered across the whole environmental gradient, differ strongly in their intraspecific responses to different drivers of change, in line with other studies^6,39^. Intraspecific changes in SLA are especially strongly associated with the successional gradient, whereas intraspecific changes in LPC are very weakly associated with succession, especially in OAK (Fig. 3). Difference between sites in the availability of nutrients, especially P, may be a stronger driver of variation in this leaf chemical trait than is succession^24^, especially on the nutrient poor soil of OAK (Supplementary Table S2).

### Changes in functional composition with succession

Our results show a directionality of changes in functional composition of sapling communities during secondary succession in TDF associated with changes in environmental conditions (canopy openness and temperature, Figs 1 and 2). However, the trends are highly dependent on the forest type, the trait and the ecological scale (intra- or inter-specific) considered.

The main trend that we found during succession was the decrease in deciduousness, observed both through the single-trait and the multi-trait analyses, which is consistent with patterns observed in Mexican TDF^20,21^. In the open and hot early stages of succession^18^ (Supplementary Table S2), deciduousness increases survival during the dry season^40^. There was also a shift from conservative towards acquisitive strategies, consistent with our expectation and with other studies in Costa Rican TDF^24,25^. This suggests that the high temperatures and irradiance of the early successional stages limit resource acquisition more than the low light of the later stages^23^. However, not all traits of the leaf economics spectrum showed this trend, and for the ones that did (leaf thickness, SLA and LNC), the contribution of species turnover and ITV differed. An increase in LNC was observed through both species turnover and ITV in MIX. An increase in SLA, consistent with the conservative to acquisitive trend, was only observed through ITV. The importance of ITV for SLA suggests a high plasticity, allowing saplings to respond quickly to rapid environmental changes^41^ such as those occurring during succession. A high ITV could also explain why previous studies of functional trait changes in TDF succession that used mean values per species (and therefore did not account for ITV) found conflicting trends: some found an increase^24–26^, while others found a decrease^20^ or no significant trend^23^ in SLA values with succession. Because of the high ITV of SLA, the choice of samples used to calculate the species mean in those studies might induce a bias to the overall trend observed during succession. For leaf density in MIX, there were contrasting trends through species turnover and ITV, as shown by their negative covariance (Fig. 3): density increased during succession through species turnover (both in the multi-trait and single-trait analyses) and decreased through ITV (multi-trait analysis). Such opposing patterns, also found across environmental gradients in temperate forests and grasslands^12,14,34^, support the hypothesis that trait values can respond differently to environmental changes depending on the ecological scale considered^6^.

Changes with successional age were weaker but changes with soil were stronger in OAK than in MIX (Supplementary Table S4). One explanation may be that the chronosequence is shorter in OAK, which may make directional changes in community trait values more difficult to observe, especially if successional processes occur more slowly under its harsher environmental conditions (higher temperatures and canopy openness, and poorer soil (Supplementary Table S2)). These harsher environmental conditions in OAK may themselves be another direct explanation. In this context, as observed in drier TDF in Mexico^23^, the main change during succession was a decrease in the proportion of legumes and their associated functional traits LNC and bipinnate leaves^33^. Legumes have several features that make them more tolerant of dry conditions. They have lower water use, related to their lower proportion of sapwood^42^. Their compound leaves favour convective over transpirational cooling and give them the ability to fold their leaflets or drop them separately thus avoiding desiccation^21,40^. Many legume species can also fix N, which confers an advantage on poor soils and often in early succession^43^.

### Intraspecific changes with ontogeny and environment

Intraspecific trait variation observed at the scale of individual saplings was driven more by fine-scale environmental factors (light and forest type) than by ontogeny. Traits associated with strategies (leaf economic spectrum and tolerance of hot and dry conditions) showed well-coordinated responses to environmental conditions, with conservative and drought-tolerance trait values more common in high light conditions. This trend was observed in the changes both with CII and with forest type (OAK being a more open and hot environment). This result is consistent with our observation at the community scale (for fewer traits) that in TDF high light levels are more of a constraint for resource acquisition, because of the hot and dry conditions, than an opportunity^23^.

The intraspecific ontogenetic changes were much less coordinated between traits; only three traits associated with the leaf economics spectrum (leaf thickness, SLA and LCC) showed significant changes from acquisitive to conservative values with increasing H/Hmax. These differences in responses of leaf economics spectrum traits support the hypothesis that they differ in sensitivity to ontogeny^6^. Although the change in resource acquisition strategies with ontogeny that we observed for some traits has been found in other biomes (temperate forests^15,28–30^ and tropical wet forests^31,32^), the underlying mechanisms are still poorly understood^29^. Several hypotheses can explain the trend from acquisitive to conservative trait values with ontogeny over the whole life of a tree: (1) the increase in radiation, wind exposure and physical abrasion experienced by taller trees can be an ultimate ecological cause for natural selection of more conservative strategies in later ontogenetic stages^28,32^, and (2) water transport limitation due to gravity can limit leaf extension in taller trees, leading to decreased SLA and increased leaf thickness^28,44^. A study in a temperate forest^45^ has shown that leaf thickness, SLA and LCC change linearly with ontogeny during the life of a tree, which can explain our result over a smaller ontogenetic window (sapling stage).

A tree growing in the successional TDF of our study is likely to experience opposed drivers of change for some traits (SLA and leaf thickness): environmental changes associated with succession driving a trend from conservative to acquisitive values, while ontogeny and the increase in crown illumination drive a change from acquisitive to conservative values. This suggests that the interaction of these different drivers may have a more complex effect in TDF than in tropical wet forests, where both succession and ontogeny drive a change from acquisitive to conservative strategies^20,31^. However, the ontogenetic window that we considered does not cover the whole life span of trees and does not extend to the reproductive stage, and although SLA and leaf thickness have been shown to follow linear trends with ontogeny in other forest biomes^45^, this remains to be tested for TDF. Further studies considering the response of a wider range of ontogenetic stages to environmental changes during succession in TDF and/or monitoring individuals through time are recommended.

### Conclusion

Our results show the importance of the ecological scale considered for the response of plant strategies and functional traits to environmental changes. Traits that are coordinated at a global scale within plant strategies (leaf economics spectrum and tolerance of hot and dry conditions) showed a decoupling of their response to environmental gradients at different ecological scales (intra- and inter-specific) when considered for the whole community, as well as their response to ontogeny at the scale of individual trees. The intraspecific response to environmental heterogeneity at an individual tree scale (crown illumination) was, on the contrary, well-coordinated for traits associated with the same plant strategy. Selecting the right traits and the right sampling design (with effort devoted to intraspecific sampling) to study a given ecological process at a given scale is therefore crucial but complex, and further studies are needed to better inform this choice.

## Methods

### Study area and chronosequences

The study was conducted in Sector Santa Rosa (10.84°N, 85.62°W), Area de Conservación Guanacaste, Costa Rica. The mean annual rainfall is 1765 mm (30 years average^24^), with a strongly seasonal distribution (the December to mid-May dry season has little or no rain) and high inter-annual variation. The vegetation type is TDF, mostly secondary forests on land previously used for crop and cattle farming^37^. We collected data from 12 plots (each 20 × 50 m) forming two chronosequences spanning respectively 12–37 and 19–67 years: six plots in the forest type dominated by oaks (*Quercus oleoides*) (OAK) and six in the forest type with a more even mixture of species (MIX)^37^. Besides having distinct tree compositions, these forest types are located on different soils (Supplementary Table S2): OAK is on nutrient-poor soil on volcanic pumice and MIX on clayey volcanic soils^37^. They therefore have distinct soil chemical properties^37^ and soil fungal communities^46^.

### Sampling design and trait measurements

We identified all saplings (1–4 m high and <7 cm diameter at breast height, DBH) of tree species in a 5 × 50 m plot located along the centre line of each of the 20 × 50 m plots (henceforth “plot” refers to the 5 × 50 m area). All sampled saplings were assigned to height classes of 50 cm intervals using a graduated stick, and their crown illumination index (CII)^47^ was assessed visually by two observers.

In June and July 2014, we measured the following leaf traits associated with (1) resource economics strategies: specific leaf area (SLA), nitrogen (LNC) and phosphorus (LPC) mass-based concentrations (high values for acquisitive strategies), and leaf thickness, density, dry matter content (LDMC) and mass-based carbon concentration (LCC) (high values for conservative strategies)^3,21^, (2) tolerance of dry and hot conditions indicated by leaf compoundness (three classes), pubescence (binary), phenological habit (three classes), density and LDMC^1,23,40^. We also measured (3) leaf area (LA) and petiole length due to their roles in light acquisition and heat regulation^11^, (4) the stoichiometric ratios C/N and N/P for their linkage to resource acquisition strategies and nutrient limitation^33^, and (5) membership of the Fabaceae (legumes) because their low water usage and their capacity to fix N suggest that they can be considered as a distinct functional group^33,42^. Standard protocols were used for these measurements (Supplementary Methods). To fully account for ITV, we measured traits on all saplings in the plots, with the exception of cases when the number of saplings per height class, per species and per plot exceeded six (6.5% of saplings; the sampled saplings were chosen randomly), and when the absence of a sufficient number of leaves prevented measurement (5.6% of leaves could not be sampled). For calculation of community scale values, non-measured leaf trait values were extrapolated (Supplementary Methods).

### Measurements of environmental factors

The successional age of each plot (i.e. age since the beginning of succession) was obtained from Powers *et al*.^37^, the basal area of adults calculated from the DBH of all trees ≥10 cm measured in 2014^24^, and the basal area of saplings from the DBH of all saplings taller than 1.3 m surveyed in our study. For each plot, during the wet and the dry season of 2014 we measured microclimatic factors expected to change with the development of canopy cover during succession: mean air diurnal temperature (henceforth “air temperature”), canopy openness and soil moisture (Supplementary Methods). Data on soil physical and chemical properties from Powers *et al*.^37^ were used to check that their variation among plots corresponds with site parent material and weathering status rather than changes with succession.

### Statistical analysis

All statistical analyses were performed in R 3.2.2^48^ using the packages ADE4^49^ and NLME^50^.

At the community level, we combined two approaches to study changes in trait values with succession: multi-trait analyses to understand strategies and trade-offs, and single-trait analyses to see if the responses to environmental drivers and across ecological scales were consistent among correlated traits. For the multi-trait analyses, we used the RLQ method on all traits and environmental factors (all microclimatic factors, successional age, adult basal area, and soil pH and clay concentration). For soil factors, we choose pH because it correlates well with other variables of soil fertility^24^ and clay concentration because it affects soil water storage. Briefly, RLQ analysis is a multivariate technique that estimates trait-environment relationships by finding axes that maximize the squared cross-covariance of linear combinations of environmental factors and traits^51^. The resulting coefficients obtained for each variable (traits and environmental factors) are used to represent the trait-environment relationship graphically. The analysis also gives the relative contribution of each trait and each environmental variable to each axis. Performing two partial RLQs^52^, controlling for species turnover and ITV, allowed separation of these two components of the trait-environment relationships. These analyses were conducted for each forest type separately to detect the differences between them.

For the single-trait analyses, we used the method proposed by Lepš *et al*.^12^ to differentiate the response of community trait values to successional age resulting from species turnover from that resulting from ITV. For each trait and each plot, we calculated two types of community weighted mean value (a) using a mean trait value *per species* and *per plot* (specific mean): changes in this value among plots reflect the effect of both species turnover and ITV, and (b) using a mean trait value *per species* calculated *across plots* of the same forest type (fixed mean): changes in this value among plots are only due to species turnover. (c) The difference between these two means (specific – fixed) gives the part that is only due to ITV. For each trait, we fitted a linear regression for each of these three values. This gives the slope and significance of the relationship between successional age and the changes in community trait value due to (a) both species turnover and ITV, (b) species turnover only and (c) ITV only, respectively. We then expressed the explained sum of squares (SS) of regression (b) (and of (c)) as a percentage of the total SS of regression (a) to get the contribution of the relationship between successional age and the community trait value that is only due to species turnover (and, likewise, only due to ITV) to the total variation of community trait values among plots. The contribution of the covariation between ITV and species turnover is obtained by expressing SS(a)-SS(b)-SS(c) as a percentage of the total SS. This expression is positive if the parts due to species turnover and ITV vary in the same direction, negative otherwise. These analyses were conducted for each forest type separately, using the trait.flex.anova function^12^.

Categorical traits did not, or very marginally, vary within species, either because the trait is inherent to the species (legumes or not), the method of data collection only considered a single value per species (phenological habit), or recorded values varied very little within species (leaf compoundness and pubescence). To assess correlation between the community values for these traits and successional age, we used quasi-binomial generalized linear models (to account for overdispersion) with a logit link (GLM). We included successional age, forest type and their interaction as explanatory variables and did a backward selection.

To test for intraspecific trait changes with ontogenetic stage and environment at the scale of individual saplings, we used the ratio between the height of the individual and the maximum height of the species (H/Hmax) as a proxy for ontogeny, which accounts for differences in maximum height among species. We chose a proxy based on height because saplings have not yet reached the canopy, and hence the asymptote of the ontogeny-height relationship. Height was taken as the middle value of the 50 cm height class to which each individual was assigned. Hmax was calculated from the height of the five tallest trees per species encountered in 60 plots in the same region^37^. To test the effect of the light environment, we used CII. For each trait, we modelled its log-transformed mean value per individual as a function of H/Hmax, forest type and their interaction (to test if the effect of ontogeny was dependent on the forest type), and CII, using linear mixed models. We included species as a random factor, both on the intercept of the model and on the slope of H/Hmax (to test if the effect of ontogeny was dependent on the species). Although crown illumination is expected to increase with increasing size, in our dataset the correlation between H/Hmax and CII is low (Kendal rank-order correlation, *Tau* = 0.077, *P* = 0.033). This allowed us to include both H/Hmax and CII in the models to disentangle changes with ontogeny and with light environment. We used the Akaike information criterion to select the structure of the random part of the model and the variables retained in the fixed part. Only species for which we had Hmax, CII and at least one individual sapling in two different height classes of the same forest type were included.

### Availability of data

Data^53^ are available from the Dryad Digital Repository: 10.5061/dryad.5535h.

## Electronic supplementary material

Supplementary information
