# Supplementary material for: Contrasting patterns of leaf trait variation among and within species during tropical dry forest succession in Costa Rica
Source: Sci Rep. 2018 Jan 10;8:285. doi: 10.1038/s41598-017-18525-1 (PMC5762662; doi:10.1038/s41598-017-18525-1)
Supplement: Supplementary file 1 — Supplementary information [file 41598_2017_18525_MOESM1_ESM.pdf]

Supplementary information

Contrasting patterns of leaf trait variation among and within species during tropical dry forest succession in Costa Rica

Géraldine Derroire, Jennifer S. Powers, Catherine M. Hulshof, Luis E. Cárdenas Varela and John R. Healey

Supplementary methods. Detailed methods for measurements of traits and environmental factors.

Plot ages

The successional age of each plot (*i.e.* age since the beginning of succession) was obtained from the estimates of Powers et al.[1], which were based on satellite images, assessment of tree age, local knowledge and stable isotopic composition of soil carbon. The ages of plots were 12, 18, 25, 28, 32 and 27 years in the forest type dominated by oak (OAK), and 19, 20, 22, 30, 42, 67 years in the mixed-species forest type (MIX).

Leaf trait measurements

Leaf traits were measured using standard protocols[2,3]. Three leaves were measured per individual except in the rare cases when the tree had an insufficient number of leaves. Because the saplings were in the understorey, we did not measure sun leaves, but instead selected three leaves that were young, fully expanded with no visible damage and located in the upper part of the sapling crown. Twigs were collected in the early morning using a tree pruner, stored in vials filled with distilled water and placed in a cool bag for transportation to the laboratory. They were stored in a refrigerator and processed on the same day. On fresh leaves, we measured leaf fresh mass (g), leaf thickness ("Thickness") (mm) and petiole length ("Petiole") (mm), and visually determined leaf compoundness (simple, unipinnate and bipinnate) and leaf pubescence (binary) with a magnifier. Leaf area (LA, cm²) was obtained by scanning the leaves and using the pixel counting software ImageJ[4]. Leaves were then oven-dried at ~60 °C for at least 72 h and re-weighed. Specific leaf area (SLA, cm² g^{-1}) was calculated as LA/dry mass, leaf dry matter content (LDMC, mg g^{-1}) as dry mass/fresh mass and leaf density ("Density") (g cm^{-3}) as dry mass/(LA x thickness). Samples of 3 g of dried leaves composed of several leaves of each individual were shipped to Bangor University for chemical analyses. Leaves were ground in a mill. Leaf C (LCC, mg g^{-1}) and N (LNC, mg g^{-1}) concentration were measured in ~0.05 g of leaf matter by combustion analysis using a CN Elemental Analyser (LECO Truspec, LECO Corporation, Michigan, USA) calibrated with certified plant standards (Orchard Leaves Part No. 502-055, LECO Corporation, Michigan, USA). Leaf P concentration (LPC, mg g^{-1}) was measured by absorbance at 820 nm using a spectrophotometer (Epoch, Biotek, Vermont, USA) on ~0.2 g samples previously ashed at 500 °C and extracted in chloridric acid. We then calculated the stoichiometric ratios of C⁄N and N⁄P. All traits were measured on leaves including petiole and rachis because we considered them to be part of the leaf construction cost. The leaf phenological habit of each species was assigned to one of three categories (deciduous, semi-deciduous or evergreen) using data from Powers and Tiffin[5] supplemented by information obtained from an expert on the local flora (D. Perez Avilez personal communication).

To fully take into account ITV, we measured traits on all saplings in the plots, with the exception of cases when the number of saplings per height class, per species and per plot exceeded six (6.5% of saplings were not measured for this reason; the measured saplings were chosen randomly). In a few cases (5.6% of leaves), the absence of a sufficient number of leaves prevented measurement. For calculation of community scale values, non-measured

leaf trait values were extrapolated by taking the mean values for the leaves of, by order of priority, (1) the same individual (when less than three leaves could be sampled), (2) all individuals of the same species, height class and plot, (3) all individuals of the same species and plot. If none of the extrapolations were possible, values were considered missing (0.5% of leaves).

Measurement of environmental factors

Air temperature was measured with one data logger (i-button DS1921G, Maxim Integrated, California, USA) located in the centre of each plot at 50 cm above the soil surface. Measurements were taken simultaneously in all plots, every hour during 8 and 11 consecutive days in the dry season (May) and in the wet season (July), respectively. The measurements taken between 5 am and 6 pm were averaged to give the mean diurnal air temperature for each season. To estimate light conditions, we used 10 hemispherical photographs taken every 5 m on the central line of each plot in the dry season (April) and in the wet season (July). The photographs were taken at 1.5 m above the soil surface using a Nikon Coolpix 4500 camera with a fish-eye lens mounted on a tripod following standard protocols[6]. They were analysed with the software HEMIv9 (Delta-T Devices, Cambridge, UK) and the values for the 10 photographs averaged to give the canopy openness (proportion of visible sky) for each season. Soil moisture was measured in the dry (May) and the wet season (November) with two measurements at eight locations within each plot, at a depth of 10 cm, using a soil moisture sensor (SM150-UM-1, Delta-T Devices, Cambridge, UK) and averaged to give one value per plot per season.

The soil physical and chemical variable values were taken from a previous study[1]. In brief, bulk density and soil elemental concentrations were obtained from volumetric samples, soil pH was measured in water and percentages of sand, silt and clay were determined with the hydrometer method. Total elemental concentrations of Al, B, Ca, Cr, Cu, Fe, K, Mg, Mn, Na, Ni, P and Zn were quantified with inductively coupled plasma emission spectroscopy. Total C and N were measured with a COSTECH Elemental Analyzer (Costech Analytical Technologies, California, USA).

References

1. Powers, J. S., Becknell, J. M., Irving, J. & Perez Aviles, D. Diversity and structure of regenerating tropical dry forests in Costa Rica: Geographic patterns and environmental drivers. *For. Ecol. Manag.* **258**, 959-970 (2009).
2. Cornelissen, J. H. C. *et al.* A handbook of protocols for standardised and easy measurement of plant functional traits worldwide. **51**, 335-380 (2003).
3. Perez-Harguindeguy, N. *et al.* New handbook for standardised measurement of plant functional traits worldwide. **61**, 167-234 (2013).
4. Abramoff, M. D., Magalhaes, P. J. & Ram, S. J. Image Processing with ImageJ. **11**, 36-42 (2004).
5. Powers, J. S. & Tiffin, P. Plant functional type classifications in tropical dry forests in Costa Rica: leaf habit versus taxonomic approaches. *Funct. Ecol.* **24**, 927-936 (2010).
6. Newton, A. C. *Forest Ecology and Conservation - A handbook of techniques.* (Oxford University Press, 2007).

Supplementary Table S1. Mean values of leaf functional traits per species. The meanings of the trait abbreviations are given in Appendix S1.

Species (number of sampled individuals)	Family	Thick-ness (mm)	Petiole length (mm)	Compoundness	Pubescence	LA (cm²)	SLA (cm² g^{-1})	LDMC (mg g^{-1})	Density (g cm^{-3})	Phenological habit	LPC (mg g^{-1})	LCC (mg g^{-1})	LNC (mg g^{-1})	C/N	N/P
Acacia collinsii (101)	Fabaceae (Mimos.)	0.105	20.85	bipinnate	No	67.563	182.778	480.11	0.57	deciduous	1.24	518.82	28.08	18.75	24.82
Acosmium panamense (3)	Fabaceae (Papilio.)	0.162	25.81	unipinnate	No	107.783	230.612	319.93	0.27	semi-dec.	1.09	518.00	32.28	16.24	30.38
Alibertia edulis (76)	Rubiaceae	0.204	5.60	simple	No	37.711	125.423	365.84	0.41	evergreen	1.06	530.45	15.50	35.05	16.73
Alophyllus occidentalis (4)	Sapindaceae	0.209	54.68	unipinnate	Yes	119.578	222.707	281.11	0.22	deciduous	1.93	490.75	36.15	14.11	19.49
Ardisia revoluta (20)	Primulaceae	0.286	7.78	simple	No	78.593	126.112	266.56	0.30	evergreen	1.35	495.95	12.88	39.33	10.40
Ateleia herbert-smithii (1)	Fabaceae (Papilio.)	0.161	52.32	unipinnate	Yes	146.742	197.364	273.84	0.32	deciduous	1.35	517.00	32.85	15.74	24.35
Bauhinia ungulata (9)	Fabaceae (Caesalp.)	0.158	11.55	simple	Yes	23.956	213.716	433.41	0.31	deciduous	1.40	537.11	26.93	20.26	19.96
Bursera simarouba (17)	Burseraceae	0.195	110.53	unipinnate	Yes	318.772	213.091	232.33	0.25	deciduous	1.43	475.76	23.01	20.96	16.74
Bursera tomentosa (25)	Burseraceae	0.193	42.05	unipinnate	Yes	98.903	222.610	290.07	0.26	deciduous	1.31	498.52	18.55	27.30	16.28
Calycophyllum candidissimum (4)	Rubiaceae	0.173	5.80	simple	Yes	50.236	318.692	179.09	0.20	deciduous	1.42	482.75	19.62	24.80	13.77
Capparis frondosa (9)	Capparaceae	0.175	32.11	simple	No	82.089	127.803	442.12	0.45	evergreen	0.73	478.78	30.36	15.88	45.93
Capparis indica (5)	Capparaceae	0.239	7.49	simple	No	31.934	102.254	416.68	0.43	evergreen	0.70	454.60	33.13	13.98	56.38
Casearia arguta (1)	Salicaceae	0.137	2.46	simple	No	30.206	280.265	282.00	0.26	deciduous	1.41	485.00	23.46	20.68	16.67
Casearia corymbosa (13)	Salicaceae	0.149	2.23	simple	No	46.594	212.934	297.36	0.33	deciduous	1.74	485.00	26.01	18.98	15.82
Casearia sylvestris (10)	Salicaceae	0.150	3.13	simple	No	17.054	186.192	357.78	0.37	evergreen	1.30	497.00	30.66	16.98	24.49
Castilla elastica (13)	Moraceae	0.144	8.89	simple	Yes	161.371	488.066	221.65	0.15	semi-dec.	2.55	463.77	42.43	11.01	16.93
Chomelia spinosa (1)	Rubiaceae	0.169	7.50	simple	Yes	27.446	249.634	243.39	0.24	deciduous	1.08	517.00	25.77	20.07	23.89
Cochlospermum vitifolium (14)	Bixaceae	0.155	169.23	simple	Yes	154.852	171.641	280.31	0.39	deciduous	1.46	522.79	21.09	25.07	15.89
Cordia gerascanthus (3)	Boraginaceae	0.184	11.55	simple	Yes	50.842	236.760	240.92	0.23	deciduous	1.46	441.00	27.18	16.53	18.59
Cordia panamensis (3)	Boraginaceae	0.226	11.92	simple	Yes	140.469	200.219	368.46	0.24	deciduous	1.42	466.67	24.07	19.52	18.43
Cornutia grandifolia (1)	Lamiaceae	0.182	2.74	simple	Yes	49.408	308.369	188.35	0.18	deciduous	2.07	495.00	35.10	14.10	16.97
Crescentia alata (1)	Bignoniaceae	0.208	85.30	unipinnate	No	22.585	186.914	298.98	0.26	semi-dec.	1.37	485.00	27.43	17.68	20.05
Dilodendron costaricense (1)	Sapindaceae	0.120	85.69	bipinnate	Yes	421.057	173.939	474.04	0.49	deciduous	0.95	541.00	18.02	30.03	18.93
Diospyros salicifolia (11)	Ebenaceae	0.206	4.71	simple	Yes	27.172	143.419	388.04	0.36	deciduous	1.24	487.73	19.31	25.70	17.30
Erythroxylum havanense (17)	Erythroxylaceae	0.185	2.71	simple	No	13.793	197.601	355.85	0.28	deciduous	1.26	481.76	27.94	17.30	22.73
Eugenia monticola (7)	Myrtaceae	0.180	2.26	simple	No	5.792	194.534	333.50	0.29	deciduous	1.39	536.43	19.44	27.63	15.09
Eugenia salamensis (1)	Myrtaceae	0.217	2.05	simple	Yes	135.639	139.735	357.14	0.33	deciduous	1.45	517.00	14.94	34.62	10.33
Euphorbia schlechtendalii (19)	Euphorbiaceae	0.140	18.18	simple	No	3.602	345.826	257.80	0.22	deciduous	2.26	506.95	22.73	22.58	10.33
Genipa americana (5)	Rubiaceae	0.212	4.62	simple	Yes	566.290	159.599	299.39	0.30	deciduous	1.22	505.60	21.02	24.41	17.51
Gliricidia sepium (10)	Fabaceae (Papilio.)	0.227	28.70	unipinnate	Yes	142.908	183.376	228.63	0.25	deciduous	1.79	505.10	33.61	15.08	19.66
Guazuma ulmifolia (4)	Sterculiaceae	0.146	10.14	simple	Yes	35.019	333.078	255.00	0.21	deciduous	1.36	475.00	25.95	18.82	19.46
Guettarda macrosperma (14)	Rubiaceae	0.142	12.17	simple	Yes	44.818	330.995	270.56	0.24	deciduous	1.16	486.21	23.05	21.71	21.05
Haematoxylum brasiletto (8)	Fabaceae (Caesalp.)	0.139	4.30	simple	No	3.391	235.927	357.99	0.32	deciduous	1.48	509.38	22.29	23.24	17.69
Hirtella racemosa (39)	Chrysobalanaceae	0.157	2.48	simple	No	14.128	149.284	468.53	0.45	evergreen	0.77	493.87	13.30	37.44	19.32
Jacquinia nervosa (2)	Primulaceae	0.282	4.47	simple	No	7.809	90.654	431.80	0.40	deciduous	0.21	503.00	11.00	47.16	53.98
Karwinskia calderonii (1)	Rhamnaceae	0.148	4.93	simple	No	7.474	224.427	404.13	0.31	deciduous	1.42	510.00	25.00	20.40	17.59

Species (number of sampled individuals)	Family	Thick-ness (mm)	Petiole lenght (mm)	Compoundness	Pubescence	LA (cm²)	SLA (cm² g^{-1})	LDMC (mg g^{-1})	Density (g cm^{-3})	Phenological habit	LPC (mg g^{-1})	LCC (mg g^{-1})	LNC (mg g^{-1})	C/N	N/P
Lonchocarpus minimiflorus (4)	Fabaceae (Papilio.)	0.183	20.49	unipinnate	Yes	54.405	210.065	358.23	0.28	deciduous	1.32	458.00	30.35	15.37	24.92
Lonchocarpus rugosus (4)	Fabaceae (Papilio.)	0.169	38.01	unipinnate	Yes	130.420	234.619	365.92	0.26	semi-dec.	1.36	503.25	29.93	17.09	22.78
Luehea candida (17)	Malvaceae	0.163	4.20	simple	Yes	75.304	379.104	324.29	0.18	semi-dec.	2.01	500.18	26.07	19.95	13.14
Luehea speciosa (24)	Malvaceae	0.162	3.80	simple	Yes	47.935	270.642	422.62	0.24	semi-dec.	1.43	500.38	27.22	18.62	20.03
Mabea occidentalis (2)	Euphorbiaceae	0.162	6.77	simple	Yes	15.709	134.664	436.56	0.47	evergreen	1.68	548.00	22.19	24.70	13.25
Machaerium biovulatum (4)	Fabaceae (Papilio.)	0.139	36.27	unipinnate	Yes	122.095	201.581	405.79	0.36	deciduous	1.22	490.50	28.33	17.36	24.24
Malvaviscus arboreus (26)	Malvaceae	0.231	46.37	simple	Yes	77.501	283.427	195.13	0.16	evergreen	1.66	409.50	29.69	14.30	18.44
Manilkara chicle (11)	Sapotaceae	0.216	16.40	simple	No	90.120	103.807	428.14	0.45	evergreen	0.63	510.27	15.72	33.07	28.71
Margaritaria nobilis (3)	Phyllanthaceae	0.149	3.29	simple	Yes	42.572	354.351	218.63	0.19	deciduous	1.76	472.67	32.41	14.72	18.42
Maytenus segoviarum (5)	Celastraceae	0.278	4.98	simple	No	23.376	92.268	411.21	0.40	semi-dec.	1.02	536.60	13.19	40.93	13.35
Mouriri myrtilloides (7)	Melastomataceae	0.175	1.11	simple	No	9.232	122.793	458.76	0.49	evergreen	0.72	500.14	14.86	33.94	24.28
Myconia argentea (2)	Melastomataceae	0.224	63.32	simple	Yes	187.228	113.490	335.75	0.40	evergreen	0.84	471.50	20.46	23.06	24.53
Myrospermum frutescens (3)	Fabaceae (Papilio.)	0.108	15.39	unipinnate	No	60.955	346.173	268.76	0.27	deciduous	2.10	504.67	30.69	16.65	14.73
Ocotea veraguensis (11)	Lauraceae	0.155	6.82	simple	No	24.017	178.808	417.11	0.37	evergreen	1.02	529.73	28.49	18.72	29.64
Pithecellobium dulce (1)	Fabaceae (Mimos.)	0.201	18.67	bipinnate	No	21.719	161.291	346.26	0.31	semi-dec.	1.61	476.00	33.34	14.28	20.76
Quercus oleoides (4)	Fagaceae	0.270	3.53	simple	Yes	22.791	83.723	493.80	0.45	evergreen	0.95	522.25	15.76	33.45	19.11
Randia monantha (16)	Rubiaceae	0.179	8.21	simple	Yes	27.747	267.136	293.91	0.22	deciduous	0.88	463.44	23.79	19.63	29.16
Rehdera trinervis (84)	Verbenaceae	0.261	6.43	simple	Yes	28.080	144.616	322.89	0.28	deciduous	1.05	501.71	17.23	29.60	18.83
Roupala montana (1)	Proteaceae	0.231	23.91	unipinnate	No	54.697	83.213	558.57	0.53	semi-dec.	0.35	539.00	11.29	47.75	31.92
Sapindus saponaria (5)	Sapindaceae	0.178	25.86	unipinnate	No	345.621	177.433	277.62	0.33	evergreen	2.70	512.20	33.87	15.64	13.16
Schoepfia schreberi (1)	Schoepfiaceae	0.262	2.79	simple	No	13.842	130.001	289.41	0.29	evergreen	1.75	459.00	17.62	26.05	10.04
Sebastiania pavoniana (37)	Euphorbiaceae	0.146	6.58	simple	No	30.969	277.146	283.14	0.26	deciduous	1.99	489.76	24.08	20.56	13.26
Semialarium mexicanum (66)	Celastraceae	0.261	3.19	simple	Yes	29.732	135.024	294.47	0.30	deciduous	1.15	447.18	16.02	28.31	15.08
Simarouba glauca (3)	Simaroubaceae	0.228	71.18	unipinnate	Yes	212.942	155.077	344.45	0.31	evergreen	1.03	491.00	20.12	26.02	21.89
Sloanea terniflora (2)	Elaeocarpaceae	0.170	8.42	simple	No	33.977	126.129	506.22	0.47	evergreen	0.77	500.50	13.85	36.57	18.54
Spondias monbin (2)	Anacardiaceae	0.234	46.37	unipinnate	No	179.375	208.947	211.77	0.22	deciduous	1.67	473.00	22.47	21.33	16.15
Stemmadenia obovata (1)	Apocynaceae	0.173	4.50	simple	Yes	141.603	344.964	171.30	0.17	deciduous	1.96	482.00	36.15	13.33	18.42
Swietenia macrophylla (2)	Meliaceae	0.151	74.57	unipinnate	No	269.822	189.832	350.81	0.36	deciduous	0.84	496.50	18.49	26.87	24.37
Tabebuia ochracea (17)	Bignoniaceae	0.166	147.72	unipinnate	Yes	417.076	216.145	373.81	0.30	deciduous	1.94	495.82	37.39	13.40	19.88
Tabebuia rosea (7)	Bignoniaceae	0.192	145.44	unipinnate	No	407.550	174.315	266.36	0.32	deciduous	1.75	488.57	21.59	22.95	12.84
Trophis racemosa (1)	Moraceae	0.161	5.50	simple	No	44.101	235.109	356.94	0.27	evergreen	1.29	465.00	23.17	20.07	18.01
Xylosma flexuosa (4)	Salicaceae	0.226	1.52	simple	No	9.723	115.393	419.94	0.41	evergreen	0.84	483.25	12.85	37.69	16.49
Zuelania guidonia (2)	Salicaceae	0.206	6.48	simple	Yes	75.134	198.590	330.98	0.25	deciduous	1.36	507.50	22.75	23.28	17.77
	Minimum	0.105	1.11	NA	NA	3.391	83.213	171.30	0.15	NA	0.21	409.50	11.00	11.01	10.04
	Maximum	0.286	169.23	NA	NA	566.290	488.066	558.57	0.53	NA	2.70	548.00	42.43	47.75	56.38

Supplementary Table S2. Environmental factors measured for each forest type. For correlation with successional age, the first number is the Pearson coefficient; *P*-values are given in brackets. Correlations with $P < 0.1$ are given in bold.

	Mixed forest		Oak forest	
	Mean (range)	Correlation with successional age	Mean (range)	Correlation with successional age
Successional age (years)	33.3 (19-67)		25.3 (12-37)	
Basal area adults (m^2 ha^{-1})	18.9 (13.0-27.2)	0.30 (0.566)	12.5 (5.3-20.3)	0.70 (0.122)
Basal area saplings (m^2 ha^{-1})	0.4 (0.2-0.9)	-0.54 (0.266)	1.0 (0.3-1.6)	-0.17 (0.741)
Air temperature (dry season) (°C)	31.2 (30.2-32.0)	**-0.75 (0.087)**	32.0 (31.0-33.2)	-0.33 (0.526)
Air temperature (wet season) (°C)	28.5 (27.9-29.2)	-0.61 (0.200)	29.7 (29.0-30.3)	**-0.76 (0.082)**
Canopy openness (dry season) (prop.)	0.32 (0.14-0.49)	**-0.90 (0.016)**	0.36 (0.14-0.53)	**-0.84 (0.037)**
Canopy openness (wet season) (prop.)	0.10 (0.08-0.13)	-0.72 (0.105)	0.18 (0.12-0.29)	**-0.98 (0.001)**
Soil moisture (dry season) (% vol)	19.5 (17.1-22.4)	0.32 (0.531)	13.0 (5.2-19.4)	-0.55 (0.260)
Soil moisture (wet season) (% vol)	32.4 (30.6-35.0)	0.51 (0.305)	24.1 (16.8-30.1)	-0.28 (0.585)
Soil pH	6.1 (5.7-6.7)	-0.37 (0.473)	5.8 (5.7-6.1)	-0.20 (0.706)
Soil stone content (g cm^{-3})	0.008 (0.003-0.012)	0.56 (0.253)	0.089 (0.007-0.327)	-0.41 (0.421)
Soil bulk density (g cm^{-3})	0.91 (0.85-1.03)	-0.30 (0.563)	0.80 (0.58-1.06)	0.26 (0.616)
Soil sand concentration (%)	40.0 (35.1-47.3)	0.01 (0.987)	44.4 (32.6-56.7)	0.50 (0.316)
Soil clay concentration (%)	31.8 (30.5-35.3)	-0.12 (0.815)	25.0 (16.7-33.4)	-0.34 (0.511)
Soil silt concentration (%)	28.2 (22.0-34.3)	0.04 (0.937)	30.5 (23.6-41.1)	-0.37 (0.471)
Soil Al concentration (ppm)	47709 (37940-58550)	**-0.84 (0.038)**	47319 (41671-59503)	-0.27 (0.610)
Soil B concentration (ppm)	19 (13-26)	-0.42 (0.412)	18 (15-20)	-0.04 (0.938)
Soil C concentration (%)	3.53 (2.99-4.14)	0.13 (0.804)	3.01 (2.33-3.61)	0.14 (0.785)
Soil Ca concentration (ppm)	7332 (4185-15248)	-0.31 (0.555)	4842 (1932-6337)	0.17 (0.742)
Soil Cr concentration (ppm)	17 (6-56)	-0.09 (0.863)	10 (8-15)	-0.23 (0.657)
Soil Cu concentration (ppm)	65 (44-114)	-0.39 (0.444)	50 (36-66)	-0.01 (0.981)
Soil Fe concentration (ppm)	33359 (22819-40040)	-0.66 (0.150)	32511 (24211-41916)	-0.43 (0.401)
Soil K concentration (ppm)	1205 (440-3731)	-0.20 (0.698)	512 (386-630)	-0.35 (0.497)
Soil N concentration (%)	0.31 (0.27-0.38)	0.38 (0.456)	0.24 (0.19-0.28)	0.12 (0.817)
Soil Mg concentration (ppm)	2840 (972-9939)	-0.17 (0.745)	1206 (825-1926)	0.16 (0.768)
Soil Mn concentration (ppm)	762 (366-1590)	-0.49 (0.321)	620 (208-1262)	-0.55 (0.256)
Soil Na concentration (ppm)	719 (516-913)	-0.23 (0.657)	739 (238-1204)	0.35 (0.492)
Soil Ni concentration (ppm)	15 (5-49)	-0.09 (0.864)	35 (9-149)	-0.38 (0.455)
Soil P concentration (ppm)	378 (124-1272)	-0.19 (0.715)	108 (31-213)	0.22 (0.682)
Soil Zn concentration (ppm)	54 (27-69)	-0.67 (0.145)	33 (19-51)	-0.43 (0.400)

Supplementary Table S3. Community mean leaf trait values per forest type (mean of the six plot mean values for each forest type, with each plot mean calculated from the values for each individual sapling in the plot).

Forest type	Oak forest	Mixed forest
Leaf thickness (mm)	0.175	0.186
Petiole length (mm)	19.88	22.35
Leaf area (cm^2)	58.685	92.618
Specific leaf area (cm^2 g^{-1})	170.630	230.177
Leaf dry matter content (mg g^{-1})	392.68	311.93
Leaf density (g cm^{-3})	0.41	0.28
Leaf phosphorous concentration (mg g^{-1})	1.26	1.37
Leaf carbon concentration (mg g^{-1})	507.31	480.50
Leaf nitrogen concentration (mg g^{-1})	21.66	25.45
Leaf C/N ratio	25.87	20.99
Leaf N/P ratio	19.55	21.03
Proportion of simple-leaved individuals	0.633	0.795
Proportion of pubescent individuals	0.335	0.567
Proportion of deciduous individuals	0.690	0.563
Proportion of legume individuals	0.329	0.066

Supplementary Table S4. Contribution ("Contrib.") and coefficient ("Coef.") of each leaf trait and each environmental factor to the first two axes of the among-species and within-species partial RLQs. Shading indicates traits and environmental factors that contribute more to an axis than if all traits and environmental factors contributed equally (> 4.7% for traits and > 10% for environmental factors). The meanings of the trait abbreviations are given in Appendix S1.

			Among-species RLQ				Within-species RLQ			
			Axis 1		Axis 2		Axis 1		Axis 2	
Forest type	Type of variable	Variable	Contrib.	Coef.	Contrib.	Coef.	Contrib.	Coef.	Contrib.	Coef.
Mixed forest	Trait	Thickness	1.58	-0.13	2.23	0.15	26.74	-0.52	2.33	0.15
		Petiole length	0.07	-0.03	4.8	-0.22	0.03	0.02	4.12	0.2
		Compoundness (simple)	0.42	0.07	1.62	0.14	0	0	0	0
		Compoundness (unipinnate)	0.52	-0.18	4.32	-0.51	0	0	0.01	-0.02
		Compoundness (bipinnate)	2.2	-0.77	2.29	-0.79	0	0	0	0
		Pubescence (glabrous)	9.3	0.47	7.03	0.41	0.17	0.06	0.06	-0.04
		Pubescence (pubescent)	6.56	-0.33	4.97	-0.29	0.12	-0.05	0.04	0.03
		LA	0.09	0.03	2.41	-0.16	0.01	0.01	20.62	0.45
		SLA	0.22	0.05	0.89	-0.09	19.13	0.44	1.83	-0.14
		LDMC	0.42	0.06	9.34	-0.31	3.81	-0.2	0.91	-0.1
		Density	5	0.22	0.03	0.02	5.15	-0.23	3.28	-0.18
		Phenological habit (deciduous)	11.09	-0.43	0.11	0.04	0	0	0	0
		Phenological habit (evergreen)	30.82	1.08	5.02	0.44	0	0	0	0
		Phenological habit (semi-deciduous)	0.54	-0.2	14.28	-1.02	0	0	0	0
		LPC	2.26	0.15	0.5	0.07	25.52	0.51	4.85	0.22
		LCC	0.09	0.03	4.71	-0.22	4.33	-0.21	3.48	0.19
		LNC	12.91	0.36	8.75	-0.3	5.06	0.23	17.08	-0.41
		C/N	4.57	-0.21	15.78	0.4	4.02	-0.2	6.45	0.25
		N/P	10.74	0.33	10.33	-0.32	5.89	-0.24	34.94	-0.59
		Not legume	0.04	0.02	0.04	0.02	0	0	0	0
		Legume	0.56	-0.29	0.55	-0.29	0	0	0	0
	Environmental factors	Successional age	26.41	0.51	4.22	-0.21	16.44	0.41	16.95	-0.41
		Air temperature (dry season)	16.07	-0.4	21.8	0.47	9.07	-0.3	0.05	-0.02
		Air temperature (wet season)	10.48	-0.32	9.23	-0.3	19.77	-0.44	3.25	-0.18
		Canopy openness (dry season)	20.84	-0.46	2.95	-0.17	19.67	-0.44	1.96	0.14
		Canopy openness (wet season)	12.69	-0.36	16.22	-0.4	18.62	-0.43	0.33	0.06
		Basal area adults	3.86	0.2	10.58	0.33	15.73	0.4	11.91	0.35
		Soil moisture (dry season)	2.83	0.17	30.4	-0.55	0.32	-0.06	2.8	-0.17
		Soil moisture (wet season)	6.07	0.25	0.27	-0.05	0.13	0.04	9.01	-0.3
		Soil pH	0.7	-0.08	1.72	-0.13	0.02	0.01	2.24	-0.15
		Soil clay concentration	0.04	-0.02	2.62	-0.16	0.21	0.05	51.48	0.72

			Among-species RLQ				Within-species RLQ			
			Axis 1		Axis 2		Axis 1		Axis 2	
Forest type	Type of variable	Variable	Contrib.	Coef.	Contrib.	Coef.	Contrib.	Coef.	Contrib.	Coef.
Oak forest	Trait	Thickness	1.38	0.12	11.13	0.33	8.45	-0.29	14.52	-0.38
		Petiole length	0.27	-0.05	5.64	0.24	0.96	0.1	0.79	0.09
		Compoundness (simple)	6.02	0.3	0.01	0.01	0	0	0	-0.01
		Compoundness (unipinnate)	0.05	0.08	3.43	0.65	0	-0.02	0.03	0.06
		Compoundness (bipinnate)	17.14	-0.83	1.58	-0.25	0	0	0	0
		Pubescence (glabrous)	0.18	0.05	12.37	-0.44	0	0	0	0
		Pubescence (pubescent)	0.31	-0.09	21.67	0.77	0	0	0	0
		LA	0.31	-0.06	1.86	0.14	0	0	0.78	0.09
		SLA	0.59	0.08	0.36	-0.06	23.74	0.49	18.32	0.43
		LDMC	2.42	-0.16	13.75	-0.37	0.31	-0.06	0.03	0.02
		Density	9.25	-0.3	7.62	-0.28	1.51	-0.12	0.6	-0.08
		Phenological habit (deciduous)	4.95	-0.27	3.75	0.23	0	0	0	0
		Phenological habit (evergreen)	10.48	0.61	10.57	-0.62	0	0	0	0
		Phenological habit (semi-deciduous)	0.49	0.37	0.35	0.31	0	0	0	0
		LPC	0	0	1.83	0.14	32.14	0.57	16.27	-0.4
		LCC	2.96	-0.17	0.92	-0.1	0.4	-0.06	0.04	-0.02
		LNC	8.35	-0.29	0.48	0.07	2.66	0.16	14.32	0.38
		C/N	9.15	0.3	1.93	-0.14	3.31	-0.18	13.1	-0.36
		N/P	3.92	-0.2	0.74	-0.09	26.51	-0.51	21.21	0.46
		Not legume	6.62	0.31	0	0	0	0	0	0
		Legume	15.17	-0.71	0	0.01	0	0	0	0
	Environmental factors	Successional age	17.66	0.42	3.69	-0.19	11.29	0.34	4.62	0.21
		Air temperature (dry season)	1.85	0.14	43.72	0.66	3.92	-0.2	1.12	-0.11
		Air temperature (wet season)	39.03	-0.62	1	-0.1	0.58	-0.08	0.56	-0.07
		Canopy openness (dry season)	1.64	-0.13	26.66	0.52	8.74	-0.3	12.1	-0.35
		Canopy openness (wet season)	15.95	-0.4	1.39	0.12	13.9	-0.37	3.7	-0.19
		Basal area adults	2.56	-0.16	9.41	-0.31	6.59	0.26	35.89	0.6
		Soil moisture (dry season)	10.53	-0.32	3.15	0.18	24.47	-0.49	10.47	0.32
		Soil moisture (wet season)	2.42	-0.16	0.72	0.09	18.21	-0.43	7.42	0.27
		Soil pH	3.74	-0.19	0.08	0.03	5.53	-0.24	1.28	0.11
		Soil clay concentration	4.62	-0.21	10.18	-0.32	6.77	0.26	22.85	-0.48

Supplementary Table S5. Slopes and significances (*P*-values in parentheses) of the linear regressions between successional age and the changes in community mean value of leaf traits due to species turnover only, intraspecific variation (ITV) only, and species turnover and ITV. The relationships with $P < 0.1$ are in bold. The meanings of the trait abbreviations are given in Appendix S1.

Forest type	Trait	Species turnover		ITV		Species turnover and ITV	
Mixed forest	Thickness	-0.0005	(0.319)	**-0.0004**	**(0.088)**	-0.0008	(0.190)
	Petiole length	0.0514	(0.839)	-0.0119	(0.867)	0.0395	(0.888)
	LA	0.4830	(0.453)	-0.2184	(0.560)	0.2646	(0.765)
	SLA	0.0770	(0.926)	**0.5772**	**(0.029)**	0.6542	(0.498)
	LDMC	0.8322	(0.247)	-0.1530	(0.270)	0.6792	(0.374)
	Density	**0.0013**	**(0.027)**	-0.0002	(0.373)	**0.0011**	**(0.081)**
	LPC	0.0036	(0.472)	0.0027	(0.185)	0.0063	(0.356)
	LCC	0.0715	(0.654)	-0.0766	(0.207)	-0.0051	(0.978)
	LNC	**0.1905**	**(0.061)**	**0.0432**	**(0.014)**	**0.2337**	**(0.036)**
	C/N	-0.1324	(0.174)	**-0.0313**	**(0.026)**	-0.1637	(0.118)
	N/P	**0.2095**	**(0.032)**	0.0097	(0.785)	**0.2192**	**(0.081)**
Oak forest	Thickness	0.0002	(0.866)	-0.0007	(0.135)	-0.0005	(0.770)
	Petiole length	-0.1775	(0.737)	0.0801	(0.460)	-0.0973	(0.859)
	LA	-0.0253	(0.986)	-0.0057	(0.981)	-0.0310	(0.985)
	SLA	0.3831	(0.798)	**1.1635**	**(0.059)**	1.5466	(0.377)
	LDMC	-0.2883	(0.912)	-0.1399	(0.736)	-0.4281	(0.877)
	Density	-0.0027	(0.554)	-0.0007	(0.247)	-0.0035	(0.490)
	LPC	-0.0036	(0.767)	0.0034	(0.684)	-0.0002	(0.993)
	LCC	-0.4342	(0.391)	-0.0292	(0.663)	-0.4634	(0.376)
	LNC	-0.1410	(0.494)	0.0456	(0.457)	-0.0954	(0.705)
	C/N	0.2088	(0.409)	-0.0418	(0.618)	0.1670	(0.590)
	N/P	-0.0774	(0.689)	-0.0470	(0.727)	-0.1245	(0.686)

Supplementary Figure S1. Patterns of leaf trait association. Correlation circle in the first factorial plane for the Hill and Smith analysis[1] (multivariate analysis with mixed quantitative and qualitative variables, performed on 2536 leaves collected from 851 saplings of 69 species using the function dudi.hillsmith of the package ade4[2] in R 3.2.2[3]. Arrows pointing in the same direction (respectively in the opposite direction) represent traits co-varying positively (respectively negatively). The closer two arrows are, the stronger the correlation among the traits. For quantitative traits, the longer the arrow, the stronger the correlation of the trait with the axis. The meanings of the trait abbreviations are given in Appendix S1. "Dec." stands for "deciduous".

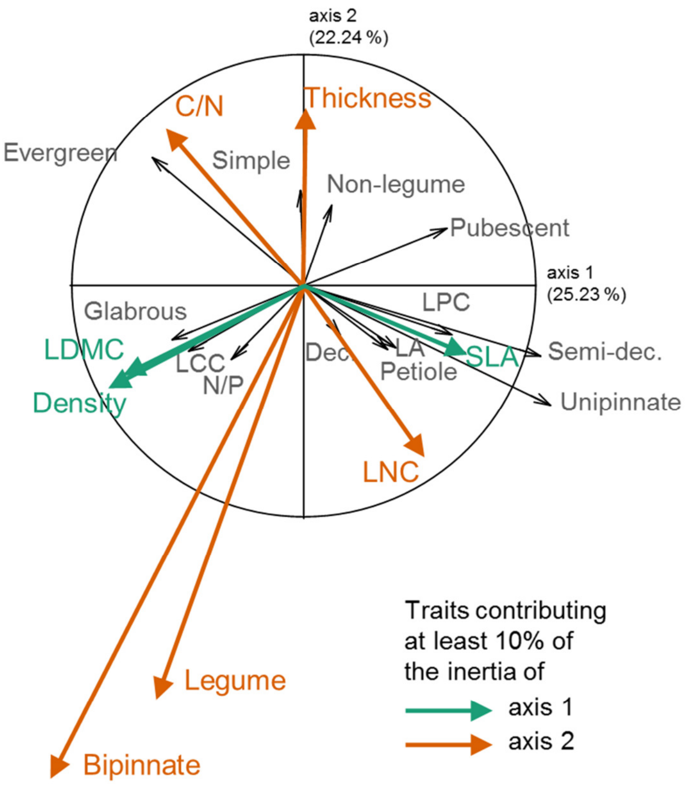

1. Hill, M. O. & Smith, A. J. E. Principal component analysis of taxonomic data with multistate discrete characters. *Taxon* **25**, 249-255 (1976).

2. Dray, S. & Dufour, A.-B. (2007) The ade4 package: Implementing the duality diagram for ecologists. *Journal of Statistical Software*, *22*, 1-20.

3. R core team. *R: A language and environment for statistical computing* http://www.R-project.org/ (2015).

Supplementary Figure S2. Partitioning of the variance of traits across scales, performed on 2539 leaves, 852 individual saplings and 69 species following the method proposed by Messier *et al.*[1]. For chemical traits, the leaf level was not considered because we only made single measurements per individual. The meanings of the trait abbreviations are given in Appendix S1.

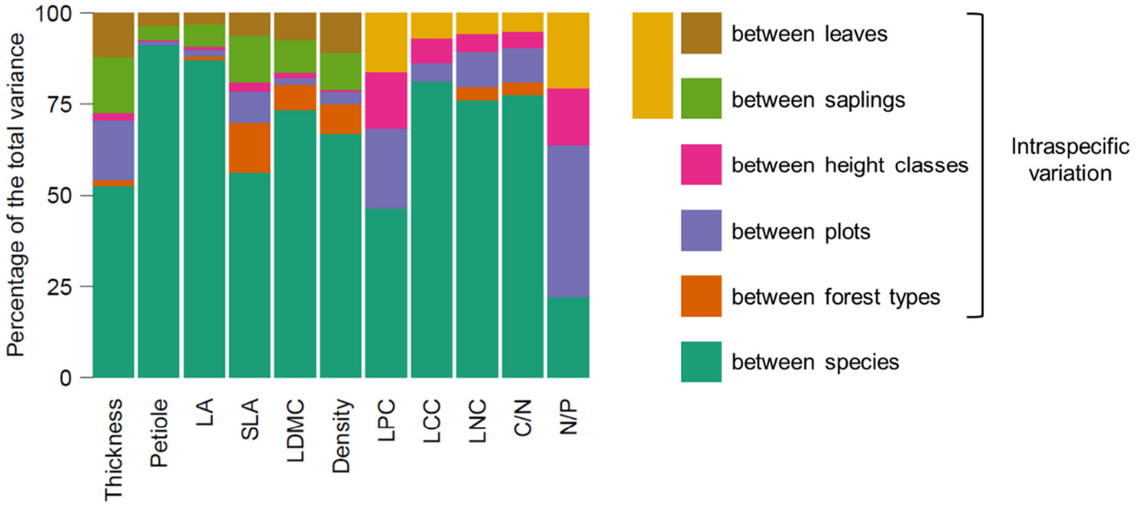

1. Messier, J., McGill, B. J. & Lechowicz, M. J. How do traits vary across ecological scales? A case for trait-based ecology. *Ecol. Lett.* **13**, 838-848 (2010).
